# Eryptosis: An Erythrocyte's Suicidal Type of Cell Death

**DOI:** 10.1155/2018/9405617

**Published:** 2018-01-03

**Authors:** Lisa Repsold, Anna Margaretha Joubert

**Affiliations:** Department of Physiology, School of Medicine, Faculty of Health Sciences, University of Pretoria, Pretoria, South Africa

## Abstract

Erythrocytes play an important role in oxygen and carbon dioxide transport. Although erythrocytes possess no nucleus or mitochondria, they fulfil several metabolic activities namely, the Embden-Meyerhof pathway, as well as the hexose monophosphate shunt. Metabolic processes within the erythrocyte contribute to the morphology/shape of the cell and important constituents are being kept in an active, reduced form. Erythrocytes undergo a form of suicidal cell death called eryptosis. Eryptosis results from a wide variety of contributors including hyperosmolarity, oxidative stress, and exposure to xenobiotics. Eryptosis occurs before the erythrocyte has had a chance to be naturally removed from the circulation after its 120-day lifespan and is characterised by the presence of membrane blebbing, cell shrinkage, and phosphatidylserine exposure that correspond to nucleated cell apoptotic characteristics. After eryptosis is triggered there is an increase in cytosolic calcium (Ca^2+^) ion levels. This increase causes activation of Ca^2+^-sensitive potassium (K^+^) channels which leads to a decrease in intracellular potassium chloride (KCl) and shrinkage of the erythrocyte. Ceramide, produced by sphingomyelinase from the cell membrane's sphingomyelin, contributes to the occurrence of eryptosis. Eryptosis ensures healthy erythrocyte quantity in circulation whereas excessive eryptosis may set an environment for the clinical presence of pathophysiological conditions including anaemia.

## 1. Introduction

Erythrocytes are derived from haematopoietic stem cells in the red bone marrow by the production of a cytokine erythropoietin produced in the kidneys [1]. The reticulocyte formed from these stem cells, following a number of differentiation steps, enters the blood stream from the bone marrow and after a few days in circulation it becomes a mature erythrocyte characterised by the absence of its mitochondria and nucleus [1].

Erythrocytes are responsible for the distribution of oxygen to body tissues and for transportation of carbon dioxide to the lungs. The pigment haemoglobin in the erythrocyte facilitates binding of oxygen and carbon dioxide and delivery of oxygen to tissues [1, 2].

Erythrocytes are constantly transported through areas of stress. These areas include the lungs where the erythrocyte is exposed to oxidative stress or through the kidneys where the erythrocyte is exposed to osmotic shock. Subsequently the erythrocyte membrane can be detrimentally affected. This may lead to the release of haemoglobin into extracellular fluid which, in turn, is filtered through the kidneys and clusters in the acidic lumen of the renal tubules ultimately leading to renal failure [2, 3].

Erythrocytes circulate the body for approximately 120 days before they are removed from the circulatory system by the process of senescence. Under certain conditions, erythrocytes undergo a form of cell death, namely, eryptosis, before they reach their full lifespan [4]. This type of cell death may be caused by an injury to the erythrocyte and may be triggered by a wide variety of factors ranging from hyperosmolarity, oxidative stress, energy depletion, heavy metal exposure, xenobiotics and antibiotics administered for various clinical conditions [2].

Characteristics of eryptosis are similar to that of apoptosis since this type of cell death also displays comparable hallmarks, namely, cell shrinkage, membrane blebbing, and exposure of phosphatidylserine on the cell membrane [5].

Eryptosis is primarily caused by an increase in cytosolic calcium (Ca^2+^) ion levels during oxidative stress and osmotic shock [6–8]. Ca^2+^ ions enter the erythrocyte through nonselective cation channels which are stimulated by prostaglandin E_2_ and by stimulators of eryptosis that trigger cell membrane vesiculation. The increase of Ca^2+^ ion levels leads to the activation of Ca^2+^-sensitive potassium (K^+^) channels, also called the Gardos channels, ultimately resulting in the loss of water as it osmotically follows the loss of potassium chloride (KCl) from the erythrocyte. Cell shrinkage in eryptosis results from activation of Ca^2+^-sensitive K^+^ channels leading to a loss of KCl from the erythrocyte ensued by the loss of water [9]. This eventually leads to the characteristic eryptotic cell shrinkage found in suicidal erythrocytes [9–12]. Cell membrane blebbing results from the activation of cysteine endopeptidase calpain, which functions by causing degradation of the erythrocyte's cytoskeleton [13–15].

With the loss of Cl^−^ ions there is a discharge of prostaglandin E_2_ which also increases the Ca^2+^ ion levels which prompts exposure of phosphatidylserine on the cell membrane [3]. Exposure of phosphatidylserine is caused by the phospholipid scrambling of the cell membrane. Once exposure of the phosphatidylserine on the erythrocyte takes place it is recognized by circulating macrophages with specific phosphatidylserine receptors and engulfed to ensure removal of the erythrocyte from the circulation [7, 8, 10].

**Figure 1:**
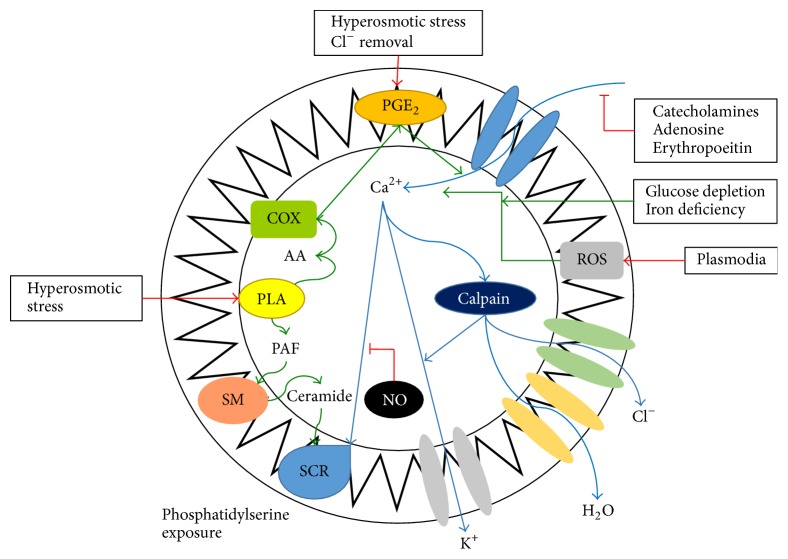
Diagram illustrating eryptosis signalling. Injury to erythrocytes activates the release of prostaglandin E_2_ (PGE_2_), which, in turn, activates the Ca^2+^ cation channels increasing the influx of Ca^2+^ ions into the erythrocyte and activating Ca^2+^-sensitive scramblase. The latter causes exposure of phosphatidylserine on the cell membrane. All this leads to the formation of the characteristics of eryptosis including membrane blebbing and cell shrinkage. NO: nitric oxide, PAF: platelet activating factor, ROS: reactive oxygen species, COX: cyclooxygenase, PLA: phospholipase A_2_, SCR: scramblase, and PGE_2_: prostaglandin E_2_ [adapted from [11]].

Cell shrinkage also causes the liberation of platelet activating factor (PAF). PAF plays a role in the control mechanism of inflammation and stimulates ceramide to be released by the disruption of sphingomyelin via the action of sphingomyelinase either present in the erythrocyte or acting from outside. As it is released into the plasma, ceramide increases the presence of the Ca^2+^-sensitive K^+^ channels (Figure 1) [2]. Ceramide is common in the presence of osmotic shock since it stimulates release of PAF by the activation of phospholipase and, as a result of the ceramide on the cell membrane, PAF produces a scrambled sarcolemma that leads to exposure of phosphatidylserine on the erythrocyte membrane. This effect of ceramide may result from the fact that ceramide induces transbilayer lipid movement [2].

Additional signalling molecules related to energy depletion further contribute to eryptosis. Janus-activated kinase 3 (JAK3), a transcription factor of critical tyrosine regulatory sites, is phosphorylated at tyrosine 980 (Tyr 980) [16, 17]. Activation of JAK3 ensuing from energy depletion results in scrambling of the cell membrane. Furthermore, casein kinase 1*α* (CK1*α*) is pharmacologically implicated in the increase of Ca^2+^ ions and the consequent stimulation of eryptosis following exposure to oxidative stress or energy depletion in erythrocytes [16, 17]. Activation of CK1*α*, resulting from pharmacological stimulation, triggers entry of Ca^2+^ into the erythrocyte through CK1*α*-opening of cation channels [16, 17].

Eryptosis functions as a protective mechanism in some cases, since it provides the erythrocyte with another form of erythrocyte cell death other than haemolysis. Haemolysis of injured or damaged erythrocytes is known to lead to the release of the contents of the erythrocyte into the blood stream, including haemoglobin that may lead to renal failure [16, 17]. Eryptosis is thus an effective form of erythrocyte cell death since it prevents erythrocytes from undergoing haemolysis and the complications attributed to this form of erythrocyte cell death. Homeostasis between eryptotic and antieryptotic mechanisms is vital to maintain normal erythrocyte count in the blood thereby preventing irregularities. Anaemia ensues in instances where increased eryptosis results in the loss of circulating erythrocytes without accompanying increase of erythropoiesis and sustaining increase of reticulocytes [16, 17].

The process of eryptosis regulation is especially complex, implicating a multitude of cellular machinery and various triggers, inhibitors, and diseases in its mechanism of action [16, 17].

## 2. Triggers of Eryptosis

Chemicals which an organism is exposed to that are foreign and therefore not inherent to the organisms are referred to as xenobiotics [16–20]. Many xenobiotics, other small endogenous molecules, and various stress-inducing procedures can trigger the mechanism of eryptosis (Table 1).

*(a) Oxidative Stress and Hyperosmolarity*. Oxidative stress and hyperosmolarity activate Ca^2+^-penetrable cation channels and Cl^−^ channels and stimulate aspartyl- and cysteinyl-proteases [21]. The loss of Cl^−^ ions stimulates release of prostaglandin E_2_, which, in turn, is the driving force behind the increase in Ca^2+^ ion levels in triggering eryptosis. Oxidative stress also activates caspases expressed by erythrocytes which promote phosphatidylserine exposure resulting in the erythrocyte being recognized and engulfed by circulating macrophages. Hyperosmolarity does not require the activation of caspases [21].

*(b) Energy Depletion*. Replenishment of glutathione (GSH) is impaired during energy depletion and therefore decreases erythrocyte antioxidant activity. Energy depletion also leads to activation of Ca^2+^-permeable cation channels in erythrocyte cell membranes triggering eryptosis by means of PGE_2_ formation [21]. Energy depletion may also influence the activity of protein kinase C (PKC) and the phosphorylation of membrane proteins leading to phosphatidylserine exposure and shrinking of the cell. In addition, activation of PKC causes an increase in the concentration of intracellular Ca^2+^ ions and the direct activation of eryptosis [21].

*(c) α-Lipoic Acid*. *α*-Lipoic acid may also cause eryptosis and activate caspase 3 [83]. In contrast to this, *α*-lipoic acid possesses an antioxidant effect in erythrocytes; in the presence of *α*-lipoic acid there is inactivation of phosphatidylserine exposure in eryptotic erythrocytes leading to the conclusion that only high doses of *α*-lipoic acid may induce eryptosis [83].

*(d) Cadmium*. Cadmium poisoning contributes to eryptosis by increasing the erythrocyte Ca^2+^ ion levels, decreasing K^+^ ion levels and shrinkage of erythrocytes. The latter explains the occurrence of anaemia in patients poisoned with cadmium [31].

*(e) Anti-A IgG*. Ca^2+^ ions influx into erythrocytes is also known to be caused by anti-A IgG antibodies leading to clearance of injured erythrocytes. This corresponds with the immune system's response to antigen A in the case of autoimmune diseases or in the case of an ABO blood transplant [28].

Erythrocytes are thus more sensitive to eryptosis than previously thought, even though they enter a hyperosmotic environment in the kidneys and are continuously subjected to oxidative stress in the lungs. The presence of any foreign substance in the blood has the potential to injure erythrocytes and to trigger eryptosis [32, 84].

## 3. Inhibitors of Eryptosis

Eryptosis may be inhibited by many substances (Table 2) and some of these substances are discussed below.

*(a) Erythropoietin*. Erythrocytes are stimulated to differentiate by erythropoietin and the latter exerts a protective function over erythrocytes and inhibits the mechanisms of eryptosis [21]. Erythropoietin functions by inhibiting the Ca^2+^-permeable cation channels which directly oppose the mechanism of eryptosis [85].

*(b) Nitric Oxide*. Nitric oxide is released from deoxygenated haemoglobin in erythrocytes and is known to inhibit eryptosis since it plays a role in vasodilation in tissues which are hypoxic by activating protein G kinase known to be of significance to erythrocyte sustenance and survival [11, 86].

*(c) Thymol*. A natural substance found in plants known to inhibit eryptosis by counteracting oxidative stress and thus the activation of Ca^2+^ cation channels is the antimicrobial agent, thymol. Not only does thymol blunt oxidative stress significantly, but it also affects the other characteristics of eryptosis including the exhaustion of energy. However, it does not inhibit the appearance of cell shrinkage [87].

*(d) Catecholamines*. Certain catecholamines such as dopamine, epinephrine, and isoproterenol are thought to suppress eryptosis by affecting the Ca^2+^ cation channels in such a way that they are unable to increase the entrance of Ca^2+^ ions; thus, eryptosis cannot occur [3]. Literature has shown that catecholamine concentrations needed to exert an antieryptotic effect are lower in the body than needed to exhibit these effects (e.g., IC_50_ of approximately 3 *μ*M for dopamine) [88]. Contradictory to this is the fact that research has shown that the treatment of certain conditions where erythrocyte toxicity takes place can be treated by dopamine to prevent the erythrocytes from undergoing eryptosis [3].

Inhibition of eryptosis is deemed important in certain clinical conditions as these substances may prove beneficial to treat patients suffering from diseases where eryptotic mechanisms are evident. Patients with sickle cell anaemia or malaria have an overstimulation of eryptosis that may result in patients becoming even more anaemic. In such cases, it can thus be beneficial to provide inhibitors of eryptosis to rectify the erythrocyte homeostasis in the blood stream.

## 4. Physiological Function of Eryptosis

Physiologically eryptosis serves as a protective mechanism by decreasing the lifespan of erythrocytes when they have been compromised due to injury or certain clinical disorders and to remove these erythrocytes from circulation. In genetic disorders such as sickle cell anaemia, glucose-6-phosphate dehydrogenase deficiency, and thalassemia, the erythrocytes have increased sensitivity to hyperosmolarity, oxidative stress, and energy depletion which decreases the erythrocyte lifespan to eliminate defective erythrocytes [3].

Natural ageing of erythrocytes is indicative of the mechanism of eryptosis since it is also hallmarked by the characteristic increase of cytosolic Ca^2+^ ions. As the erythrocyte ages, it is regularly exposed to oxidative stress, hyperosmolarity, and energy depletion and the ageing erythrocyte may lose its ability to protect itself from these harmful conditions finally resulting in the removal of these erythrocytes from the circulation by senescence [2]. The mechanism of senescence has been compared to that of eryptosis since they both share many of the same characteristics [2].

In the case of malaria, the increased incidence of eryptosis is beneficial since it limits the growth of the* Plasmodium falciparum* parasite in the erythrocyte [11]. Once the erythrocyte is infected there is an increase in oxidative stress and the resulting entrance of Ca^2+^ ions by the cation channels which activate eryptosis. Even though the parasite relies on the activation of these channels to obtain its nutrients, eventually the increased Ca^2+^ ion levels in the erythrocyte will trigger eryptosis and the subsequent removal of the infected erythrocytes from circulation [11].

An important physiological function of eryptosis is to prevent the occurrence of haemolysis and the resulting complications thereof. Haemolysis can be triggered by energy depletion, Na^+^/K^+^-ATPase defects, and leaking of the cell membrane which results in the entrance of Na^+^ and Cl^−^ ions into the erythrocyte and water along the osmotic gradient causing erythrocyte swelling. Initial entrance of Na^+^ ions can be compensated by the loss of cellular K^+^ ions. However, the increased loss of K^+^ ions will result in membrane depolarisation and subsequent entry of Cl^−^ ions [2, 3, 11]. The increase in cell volume and resulting swelling lead to erythrocyte membrane rupture and the release of unwanted erythrocyte contents, in particular haemoglobin, into the circulatory system [11].

## 5. Newborns

Foetuses and newborns have a different form of haemoglobin compared to adults since they obtain their oxygen from the placenta and not from respiration in the lungs. Foetal haemoglobin (HbF) has a higher affinity for oxygen than normal haemoglobin. This allows for the foetus to obtain enough oxygen from the placental circulation. Since foetal haemoglobin has a higher affinity for oxygen it allows for less oxygen release to the periphery and not full oxygenation of the haemoglobin in the foetal erythrocyte [11].

Foetal haemoglobin is thus not functionally suited for effective gas exchange after birth; however, foetal erythrocytes are more resistant to the removal of Cl^−^ ions, hyperosmolarity, prostaglandin E_2_, and PAF, yet it is more susceptible to oxidative stress [11]. As a result of foetal haemoglobin's sensitivity to oxygen, it promotes removal of these erythrocytes from the neonate's circulation once they have been exposed to inspired oxygen after birth [11].

## 6. Neocytolysis

Neocytolysis is the occurrence of erythrocyte cell death in newly formed erythrocytes; this form of cell death is known to be caused by a fall in erythropoietin levels [68]. Research revealed that neocytolysis may resemble the mechanism of eryptosis since mice that overexpress erythropoietin have erythrocytes which are unaffected by osmotic lysis; their erythrocytes are, however, more sensitive to elimination of Cl^−^ ions and Ca^2+^ ionophore ionomycin exposure. Erythrocytes may therefore be more sensitive to eryptosis in conditions where the erythropoietin levels are low, since erythropoietin can increase the expression of genes in progenitor cells [11].

The occurrence of neocytolysis has been observed frequently in patients at high altitudes or exposed to space flights, since these patients have high concentrations of erythropoietin and once these levels of erythropoietin are lower, the incidence of eryptosis increases. Neocytolysis can be seen as an effective feedback regulation mechanism since, during high concentrations of erythropoietin, there is upregulation in erythrocytes of proeryptotic effectors prompting removal of excessive erythrocytes. Once the concentration of erythropoietin decreases, the number of erythrocytes will decrease accordingly [11, 21, 25]. Neocytolysis contributes to the maintenance of normal erythrocyte count by selectively haemolysing young erythrocytes in response to decreased erythropoietin levels [68].

## 7. Pathophysiology behind Eryptosis

Various clinical conditions or diseases are known to cause eryptosis; the most common conditions are usually in accordance with anaemia. Recent reports have implicated eryptosis in cytostatic treatment of various malignancies [16]. The mechanism of action is not entirely understood, but it is thought to be triggered by oxidative stress and ceramide formation in lung cancer patients [89]. In diabetic patients, chronic hyperglycaemia in these patients causes stimulation of eryptosis through methylglyoxal which decreases ATP and glutathione (GSH) concentrations [16]. Haemolytic uremic syndrome is characterised by haemolytic anaemia and activates eryptosis through oxidative stress and lipid peroxidation [16]. In sepsis, erythrocytes within the circulation are exposed to the supernatant of the implicated pathogen and oxidative stress, activating eryptosis [16]. Some of these conditions are mentioned in Table 3 and are discussed below.

*(a) Renal Insufficiency*. Renal insufficiency or end-stage renal disease (ESRD) is defined as a decrease in the function of the kidneys in filtering waste products and excess fluid from the body [90]. ESRD also results in reduced production and release of erythropoietin which compromises erythrocyte production via erythropoiesis causing anaemia [16, 17, 90]. Studies have shown that the number of phosphatidylserine-exposed erythrocytes in ESRD patients is significantly higher than in healthy individuals [90]. The significant increase in eryptosis in this patient population is directly linked to the increased number of uremic toxins such as methylglyoxal in ESRD patients and treatment of ESRD with either haemodialysis or peritoneal dialysis [90].

*(b) Psychosis*. Some medications for clinical conditions may also trigger eryptosis. This includes chlorpromazine, an antipsychotic drug. A study conducted by Akel et al. [32] indicated that the concentrations of the drug given to treat patients are high enough to cause eryptosis by means of cell membrane exposure of phosphatidylserine [32]. Chlorpromazine also functions by reducing ATP levels in the erythrocyte, as well as increasing the Ca^2+^ ion levels which together lead to hallmarks of eryptosis including hyperosmolarity, glucose depletion, and shrinking of the erythrocyte [32].

*(c) Wilson's Disease*. Accumulation of Cu^2+^ ions in the liver, brain, and other vital organs of patients diagnosed with Wilson's disease results in the activation of eryptosis via ceramide formation leading to anaemia and cirrhosis of the liver [86]. Possibly the ceramide formation in these patients can also be attributed to sphingomyelinase activity in the serum which leads to increased eryptosis [25].

*(d) Malaria*.* Plasmodium falciparum*: the microorganism responsible for malaria uses eryptosis to its advantage to hide from the immune response [13]. The microorganism is able to use nonselective cation channels to obtain nutrients [13].* Plasmodium falciparum* uses Ca^2+^ ions for cation channel activation, thus obtaining required nutrients, which is present in the cell undergoing eryptosis, thereby enabling the erythrocyte to survive longer in a state of eryptosis since it decreases the cytosolic Ca^2+^ ion concentrations [13].

*(e) Sickle Cell Anaemia and Glucose 6-Phosphate Dehydrogenase Deficiency*. In diseases, namely, sickle cell anaemia and glucose 6-phosphate dehydrogenase deficiency, erythrocytes are more susceptible to eryptotic activators, for example, oxidative stress and energy depletion [3].

*(f) Iron Deficiency Anaemia*. Erythrocytes of patients with iron deficiency anaemia usually present with decreased cell volume. The latter triggers increased activation of cation channels and subsequent increased cytosolic Ca^2+^ and results in eryptosis and a shortened erythrocyte lifespan [11]. The shortened lifespan of iron-deficient erythrocytes also corresponds to increased exposure of phosphatidylserine on the surface of the erythrocyte and coincides with eryptosis [3].

*(g) Thalassemia*. Thalassemia is an autosomal recessive disease which leads to reduced and defective development of haemoglobin in erythrocytes. This leads to erythrocytes being more susceptible to oxidative stress and increases the incidence of anaemia since erythrocytes are prematurely removed from the circulation by means of eryptosis [3]. Literature has shown that insulin may protect thalassemic erythrocytes from increased oxidative effects. They endure the latter since insulin decreases the occurrence of phosphatidylserine exposure and increases glycolysis in erythrocytes which, in turn, increases the amount of ATP in the erythrocyte leading to the inhibition of PKC [91]. Since it is known that PKC directly influences eryptosis caused by energy depletion, treatment of these patients with insulin will result in inhibition of eryptosis [91].

As previously mentioned, eryptosis enables the body to rid itself of erythrocytes in a more convenient manner before the erythrocytes are removed by haemolysis. Haemolysis is known to result from erythrocytes with cell swelling, loss of potassium, and subsequent Cl^−^ ion entrance [2, 3, 11]. Swelling injures the cell membrane and leads to rupturing of the erythrocyte and the release of haemoglobin which precipitates in the tubules of the kidneys as it is filtered, leading to renal failure.

## 8. Conclusion

Eryptosis resembles the mechanism of apoptosis in nucleated cells [92, 93]. Eryptosis may also be increased in certain conditions where the structure of haemoglobin is altered such as in the case of sickle cell anaemia and thalassemia [94]. Mature erythrocytes still possess erythropoietin receptors which then influence the prevalence of eryptosis in erythrocytes through regulation of the cation channels [95].

Erythrocytes are continuously being exposed to hyperosmolarity through the formation of the kidney filtrate and oxidative stress through oxygen and carbon dioxide transport in the lungs. Even though erythrocytes are constantly exposed to extreme conditions, they manage to repair some of the damage without the presence of a nucleus or mitochondria. This may be attributed to the fact that erythrocytes contain enzymes which have a protective function. Erythrocytes can only recover from a limited period of injury until it is subjected to the fate of either senescence or haemolysis [96]. It is not clear how the erythrocytes differentiate between entering either eryptosis or haemolysis. However, it is known that erythrocytes would rather enter eryptosis, since eryptosis is a precautionary route of the body to limit premature haemolysis of injured erythrocytes thereby preventing unwanted increased haemoglobin levels [97–99]. Haemolysis can be prevented if swelling of the erythrocyte is preceded by eryptosis, thus enabling phosphatidylserine exposure and subsequent removal of the erythrocytes before haemolysis can occur [11].

In a recent literature study conducted by Lefrançais et al. [100], it was reported that the lungs can be regarded as a reservoir for haematopoietic progenitors and can reconstitute bone marrow with multiple haematopoietic lineages [100]. The haematopoietic potential of the lungs, the role they play in erythropoiesis, and the potential corresponding signalling mechanisms where eryptosis may be implicated are of significance when considering these new findings emphasized by Lefrançais et al. [100].

It can be concluded that erythrocytes undergo a more complex type of cell death than conventionally thought. The mechanism of eryptosis indicates the significant role that cation channels play in erythrocytes [82, 92]. Research on the signalling mechanisms of eryptosis is vital to further unravel the phenomena of suicidal erythrocyte death and to identify clinical conditions and substances which may trigger eryptosis or in which eryptosis may benefit the survival of the erythrocyte.

## Figures and Tables

**Table 1:** Factors that stimulate eryptosis. Activation mechanisms can occur through Ca^2+^ ion entry, ceramide formation, or adenosine triphosphate (ATP) depletion.

Stimulating factors	Activation mechanism	References
Aluminium	Ca^2+^	[22]
Amantadine	Ca^2+^	[23]
Amiodarone	Ca^2+^	[24]
Amphotericin B	Ca^2+^; ATP depletion	[25]
Amyloid	Ceramide	[26]
Anandamide	Ca^2+^	[27]
Anti-A IgG	Ca^2+^	[28]
Arsenic	Ca^2+^; ceramide; ATP depletion	[29]
Azathioprine	Ca^2+^	[30]
Bismuth chloride	Ca^2+^; ceramide	[25]
Cadmium	Ca^2+^; ATP depletion	[31]
Chlorpromazine	Ca^2+^	[32]
Ciglitazone	Ca^2+^	[33]
Cisplatin	Ca^2+^	[34]
Copper	Ceramide	[35]
Cordycepin	Ca^2+^	[36]
Cryptotanshinone	Ca^2+^	[20]
Curcurmin	Ca^2+^; ceramide	[37]
Cyclosporine	Ceramide; ATP depletion	[38]
CD95/Fas/ligand	ATP depletion	[39]
Glycophorin-C	ATP depletion	[40]
Gold chloride	Ca^2+^	[41]
Hemin	Ca^2+^; ceramide	[25]
Hemolysin	Ca^2+^	[42]
Lead	Ca^2+^	[43]
Leukotriene C	Ca^2+^	[25]
Lipopeptides	ATP depletion	[25]
Listeriolysin	Ca^2+^; ATP depletion	[44]
Lithium	Ca^2+^	[45]
Mercury	Ceramide; ATP depletion	[46]
Methyldopa	Ceramide; ATP depletion	[47]
Methylglyoxal	Ceramide	[48]
Paclitaxel	Ca^2+^; ceramide	[49]
PAF	Ceramide	[50]
Peptidoglycan	Ca^2+^; ceramide	[51]
Radiocontrast agents	Ca^2+^; ATP depletion	[25]
Retinoic acid	Ca^2+^	[52]
Selenium	Ca^2+^; ceramide	[53]
Silver ions	ATP depletion	[25]
Thrombospondin-1-receptor CD47	ATP depletion	[54]
Thymoquinone	ATP depletion	[25]
Tin	Ca^2+^; ceramide; ATP depletion	[55]
Valinomycin	ATP depletion	[7]
Vanadate	Ca^2+^	[56]
Vitamin K (3)	Ceramide	[25]
Zinc	Ca^2+^; ceramide	[57]

**Table 2:** Factors inhibiting eryptosis. Inhibition mechanism can be through the Ca^2+^ entry mechanism, ceramide formation, or ATP depletion.

Inhibiting factors	Inhibition mechanism	References
Adenosine	Ca^2+^; ATP depletion	[58]
Amitriptyline	Ceramide; ATP depletion	[25]
Caffeine	Ca^2+^; ATP depletion	[25]
Catecholamine's	Ca^2+^	[25]
Chloride	Ca^2+^	[59]
Ethylisopropylamiloride	Ca^2+^	[60]
Erythropoietin	Ca^2+^	[61]
Flufenamic acid	Ca^2+^	[62]
Quinoxalinediones (NBQX/CNQX)	Ca^2+^	[25]
Niflumic acid	ATP depletion	[25]
Nitric oxide (nitroprusside)	ATP depletion	[63]
Natriuretic peptide precursor B	ATP depletion	[25]
Resveratrol	Ca^2+^; ATP depletion	[25]
Staurosporine	ATP depletion	[64]
Urea	Ceramide; ATP depletion	[59]
Xanthohumol	Ca^2+^; ATP depletion	[25]
Zidovudine	Ca^2+^	[65]

**Table 3:** Diseases resulting in accelerated eryptosis. Activation mechanisms occur through Ca^2+^ ion entry, ceramide formation, and inhibition of ATP depletion.

Disease	Activation mechanism	References
Iron deficiency	Ca^2+^	[66]
Phosphate depletion	ATP depletion	[67]
Neocytolysis	ATP depletion	[68]
Sepsis	Ceramide formation	[69]
Haemolytic anaemia	Ca^2+^; ceramide formation; ATP depletion	[70]
Haemolytic uremic syndrome	Ca^2+^; ceramide formation	[71]
Renal Insufficiency	Ca^2+^	[61]
Malaria	Ca^2+^	[72, 73]
Sickle cell disease	ATP depletion	[74–77]
Thalassemia	ATP depletion	[77–79]
Glucose-6-phosphate dehydrogenase deficiency	ATP depletion	[80]
Wilson's disease	Ceramide formation	[35]
Anion exchanger 1 mutation	Ca^2+^	[81]
Glucose transporter 1 mutation	Ca^2+^	[82]
